# Prevalence of Use and Knowledge about Tobacco Products and Their Harmful Effects among University Students in Southern Croatia

**DOI:** 10.3390/healthcare11050771

**Published:** 2023-03-06

**Authors:** Dora Istenic, Lidia Gavic, Antonija Tadin

**Affiliations:** 1 School of Medicine, University of Split, 21000 Split, Croatia; 2 Department of Restorative Dental Medicine and Endodontics, Study of Dental Medicine, School of Medicine, University of Split, 21000 Split, Croatia; 3 Department of Maxillofacial Surgery, Clinical Hospital Centre Split, 21000 Split, Croatia

**Keywords:** smoking behavior, health risks, knowledge, university students, tobacco products

## Abstract

Understanding students’ attitudes toward smoking and tobacco products is essential for effective smoking prevention interventions. This questionnaire-based cross-sectional survey aims to determine the prevalence of use and knowledge about cigarettes, heated tobacco products, and electronic cigarettes and their harmful effects among university students. The survey was conducted using a self-administered online questionnaire among 1184 students. Questions were related to the respondents’ demographic characteristics, tobacco use patterns, and opinions about exposure to health warnings and tobacco product advertising messages. Data were analyzed using descriptive statistics and generalized linear regression analysis. The results showed that 30.2% of the students use tobacco products (74.5% smoked conventional cigarettes; 7.9% used electronic cigarettes; and 17.6% used heated tobacco products). The median (interquartile range) score of the students’ knowledge (maximum = 27) was 16 (12–22). The results showed that students from technical, social, humanities, natural, and biotechnology scientific programs had lower levels of knowledge about tobacco products and their harmful effects than biomedical students (*p* ≤ 0.001). In addition, past and current use of tobacco products was significantly associated with higher overall knowledge of tobacco products and their harmful effects (adjusted odds ratio (OR) 1.90, % confidence interval (CI) 1.09–3.31, *p* = 0.023; OR 1.41, CI 1.08–1.84, *p* = 0.011). The research findings confirm the lack of knowledge and misconceptions about the harmful effects associated with tobacco product use. They also emphasize the need for better prevention and awareness of the harmful effects of smoking on human health.

## 1. Introduction

Although the adverse health effects of tobacco product use are well known, it remains one of the most significant public health challenges in all age groups, especially among young people [1]. According to 2020 study data, the prevalence of smoking in Croatia was 36%, the third-highest tobacco prevalence in Europe [2]. As in most countries, Croatians start smoking at an average age of 18.2 years [2,3].

Starting university is perhaps one of the most challenging periods of a young adult’s life, as they are leaving their routine and building an independent lifestyle in a more liberal environment. During this time, young people are part of an at-risk group for starting to use different tobacco products [4]. Scientific studies conducted on young people confirm that individuals who started smoking at a younger age can become addicted to nicotine very quickly, smoke more cigarettes per day, and are less likely to try to quit. Smoking initiation and continuation depend not only on sociodemographic factors, culture, family history of smoking, and health literacy, but also on social, emotional, and educational challenges during schooling [1,4]. It is well known that smoking is a preventable risk factor, and the literature identifies numerous interventions to reduce smoking initiation. These measures include education through school programs, increasing product prices, warning labels on packaging, limiting product advertising, smoke-free environments, and restricting minors’ purchase of tobacco products [5]. Since most smokers start smoking before or immediately after graduating high school, this age group should be considered the primary target group for smoking prevention programs [6]. The decision to not smoke or to quit smoking is a serious matter in which educational institutions, including universities, can play an important role. Since the university environment is a significant factor in health promotion, it is crucial for smoking prevention that universities create an environment that promotes anti-tobacco attitudes and behaviors [7,8].

To support smoking prevention and promote healthy lifestyles, it is essential to understand young people’s knowledge and attitudes about tobacco product use. Numerous studies have examined the relationship between knowledge, attitudes, and smoking habits among young adults and schoolchildren, both in Europe and worldwide [4,6,9,10,11]. However, studies on habits of tobacco product use among youths and students in Croatia are rare. In 2008 and 2022, two studies were conducted only on medical students, which confirmed that over 30% of them currently smoke [11,12]. After an extensive literature review, to our knowledge, this is the first study to examine the prevalence of tobacco use and knowledge of the adverse health effects of tobacco use among medical and non-medical students in southern Croatia.

Thus, the objectives of this study were: (1) to assess tobacco product use behaviors and (2) to analyze the relationship between knowledge about tobacco products and their harmful effects and attitudes regarding tobacco product use. All of the data support research activities on the prevalence of tobacco product use, which can help clarify the current situation and develop prevention programs in academic institutions.

## 2. Materials and Methods

The questionnaire-based cross-sectional survey was conducted from November 2021 to February 2022 among the students of the University of Split in the academic year 2021/2022. The study was conducted at the Department of Restorative Dental Medicine and Endodontics, School of Medicine, University of Split, Croatia, and approved by its Ethics Committee. The working methodology was performed according to the recommendations and applicable regulations and following the institutional code of ethics.

### 2.1. Participants and Setting

The survey was based on a self-administered questionnaire created through a network survey (Google Forms). It was sent to the Students’ Association representative, who distributed it electronically to other students through the University’s Facebook group with 6579 members. Respondents were also asked to forward the questionnaire to other interested colleagues. A non-probability convenience and a snowball sampling method were used to recruit respondents. Students from the age of 18 years and older and of both genders participated in the study. A sample of 1184 students completed the survey, of which 72.3% were female and 27.7% were male. The mean age was 22.1 ± 2.6 years (minimum 18, maximum 49). Minors and individuals who did not answer the questions were not included in the study. Requirements for participation were students enrolled at the University of Split in the academic year 2021/2022 and a fully answered survey. The survey ensured anonymity, and participation was not financially supported. In addition, all participants could drop out at any time during the study.

At the University of Split, Croatia, students from 18 different programs participated in the survey: four from healthcare (medicine, dentistry, pharmacy, and health sciences) and 14 from other disciplines (economics, electrical engineering, mechanical engineering and naval architecture, humanities and social sciences, civil engineering, architecture and geodesy, Catholic theology, chemistry and engineering, kinesiology, law, natural sciences, marine biology, forensics, professional studies, and art academy).

The introduction to the survey provided participants with all relevant information and a description of the purpose of the study. Participation was voluntary and anonymous, and completion of the questionnaire was taken as consent to participate in the study. The required minimum sample size (*n* = 377) was calculated from the total number of students at the University of Split in the academic year 2021/2022 (*n* = 19,100), with a confidence interval of 95%, a margin of error of 5%, and a response distribution of 50%.

### 2.2. Questionnaire

A structured questionnaire was modified based on several studies on the same topic [3,5,8,13,14,15,16,17], consisting of four parts and 50 questions [18]. The first part included primary demographic data of the respondents (gender, age, year of study, employment of a family member in the medical field, and assessment of the family’s financial situation). The second part included ten questions about tobacco product use (tobacco product use, type of tobacco product use, duration of tobacco product use, when did they start using tobacco products, do their relatives know that they use tobacco products, do they have a desire to stop using tobacco products, smoking status of family members, and knowledge of smoke-free electronic devices such as e-cigarettes and tobacco heaters). The third section consisted of 27 questions assessing respondents’ understanding of the adverse health effects of tobacco products (14 questions), health risks associated with secondhand smoke (six questions), and knowledge of tobacco products (seven questions). Respondents could check one of the three answers offered (yes/no/do not know). Overall knowledge was calculated based on the sum of the correct answers, and a score between 0 and 27 was obtained. Thus, the higher the total score, the greater the respondents’ awareness of smoking-related diseases and secondhand smoke. The fourth part consisted of seven questions related to respondents’ opinions about exposure to health warnings and tobacco product advertising messages: have they noticed anti-smoking messages in the media, tobacco advertising in stores, use of tobacco products on television, i.e., health warnings on cigarette packets? Are they thinking about quitting smoking because of the warnings on the packages? Have they informed themselves about the dangers of tobacco? Do they think that smoking should be banned in enclosed public places, and what should be done to reduce the number of smokers?

Before the Internet survey was sent to the respondents, two dentists (university professors) reviewed the survey. They agreed with the content of the prepared questionnaire. Before distributing the questionnaire, a pilot study was conducted with 30 students (15 from medical and 15 from non-medical fields of study) to check the reliability of the questionnaire. These questionaries were not included in the primary study data. The pilot study provided evaluation of the time needed to complete the questionnaire, which was approximately 12 min. The internal consistency of the total scores yielded a Cronbach’s coefficient alpha of 0.712.

### 2.3. Statistical Analysis

Statistical Package for the Social Sciences, version 26 (SPSS, IBM Corp, Armonk, New York, NY, USA) was used for statistical data analysis. Frequencies were calculated for each categorical variable (question from the survey). The results are presented in tabular form. The Kolmogorov–Smirnov test was used to assess the normality of the distribution of responses. The association of demographic characteristics (age, gender, study filed, family members in the medical field, family’s monthly budget and smoking status) with knowledge of tobacco products and their harmful effects was tested using generalized linear model (GLM) analysis. The significance level was set at *p* < 0.05.

## 3. Results

Table 1 shows the sociodemographic data of the respondents. The study was conducted with 1184 first to sixth-year students, of whom 72.3% were women and 26.7% were men. 

Table 2 shows the results regarding the tobacco use of the respondents. Most of the respondents had never used tobacco products (64.3%); 5.6% were former users who had not used a product for at least 28 days; and 30.2% were current users of tobacco products. Although former users had the best knowledge, there was no statistically significant difference between these three groups. However, students who wanted to quit tobacco use had better knowledge and knew the difference between different tobacco products (*p* ≤ 0.001).

Of the respondents, 30 (2.6%) did not give a single correct answer, while 164 (13.9%) correctly answered on all 27 questions used to assess their knowledge about tobacco products and their harmful effects on human health (see Supplementary File, Table S1). The median (interquartile range) score of the students’ knowledge (maximum = 27) was 16 (12–22).

Table 3 shows the responses to the questions assessing respondents’ attitudes toward exposure to health warnings and tobacco product advertising messages. 

The results of regression analysis showed that students from technical, social, humanities, natural and biotechnical science have a lower level of knowledge of tobacco products and their harmful effects than a biomedical student (*p* ≤ 0.001). Former and current tobacco product usage was significantly associated with higher overall knowledge of tobacco products and their harmful effects (*p* = 0.023 and *p* = 0.011) (Table 4.)

Table 5 presents the results of adjusted and unadjusted associations of the GLM for knowledge of health risks of secondhand smoke and knowledge of tobacco products. Study of technical, social, humanities, natural and biotechnical science was significantly associated with lower overall knowledge of health risks of secondhand smoke (*p* ≤ 0.001), while studying technical science was the only program significantly associated with lower overall knowledge of tobacco products (*p* = 0.007).

Table 6 presents the results of the gender-specific GLM analysis on knowledge about tobacco products and their harmful effects. In women, former and current tobacco product usage was significantly associated with higher overall knowledge of tobacco products and their harmful effects (*p* = 0.023 and *p* = 0.021).

## 4. Discussion

The results of the study showed that university students had a moderate to low level of knowledge regarding tobacco products and their harmful effects on health, with a median score of 16 out of a possible 27 points. In the knowledge assessment questions, students showed good knowledge when answering questions about health risks associated with active smoking. Similar results were obtained in studies with student test groups in Egypt and Pakistan [19,20]. In contrast, respondents in this survey showed below-average knowledge on questions about the health risks of secondhand smoke and knowledge of the difference between tobacco products. Numerous studies worldwide demonstrate the lack of awareness of the dangers of secondhand smoke and its many health effects [21,22].

The survey results show that the knowledge of tobacco products and their adverse health effects vary among respondents from different studies and scientific fields. Students from biomedical and health sciences had the highest knowledge level, while students from technical studies had the lowest knowledge level. Students in biomedical and health science programs are the future of smoking prevention and potential leaders of such interventions, which require accurate information on this topic. Therefore, their knowledge is crucial for the national tobacco control strategy [15]. Most European medical and biomedical schools provide adequate theoretical training on tobacco culture and its health effects in their curricula; however, a much smaller proportion is provided in the form of practical training, which is crucial for developing high-quality educators [23,24,25].

Regarding knowledge levels, several previous studies have shown that active smokers have inadequate knowledge about the harmful effects of smoking, suggesting that improving knowledge levels positively impact smoking prevalence [2,26]. In this survey, no differences were found in the knowledge of respondents’ tobacco use patterns. Of the respondents, 30.2% were current users, and 70.9% started using tobacco products in high school. The prevalence of active smokers is consistent with the 2016 European Union survey, in which 35% of adults in Croatia were registered smokers [27]. Respondents in this survey smoked mainly conventional cigarettes (74.5%); 7.9% used e-cigarettes; and 17.6% used heated tobacco devices. The prevalence rate of e-cigarette use was similar to that published for the adult population in Croatia (5.8%) but much higher than the prevalence rate published in the 2017 Eurobarometer (1%) [2,28]. In addition, the prevalence of heated tobacco product use was much higher than the results of the study using Eurobarometer data from 2020 [29], which was 6.8%.

Less than half of the respondents in this survey had the attitude that smokeless devices are a better alternative to traditional smoking. However, the same respondents had a significantly higher level of knowledge and better knew the differences between cigarettes, e-cigarettes, and heated tobacco products. Nowadays, more and more young people are choosing to switch to e-cigarettes or heated tobacco products. This is thought to be due to several factors, such as a health-related desire to cut down/quit smoking and the perceived lower adverse effects of the new products, branded packaging, and lack of visual warnings. In addition, pleasure and satisfaction during use, pleasant sensory experiences, lower odor, tactile similarity to cigarettes, and psychological similarities in rituals and routines also contribute [30]. Although smokers consider the allegedly less harmful alternative tobacco products to be relatively safe, the need to educate smokers about the risks of alternatives to conventional cigarettes and to regulate the marketing and advertising of such options must be emphasized [31,32].

Respondents in this survey showed good knowledge about the harmful effects of smoking on general and oral health. The respondents showed the best knowledge on the questions about the relationship between tobacco and carcinomas, where 90.6% answered correctly. In addition, 85.5% of respondents knew that smoking causes cardiovascular disease. 91.7% of the respondents knew that smoking during pregnancy affects the child’s health, which is of great importance, because smoking parents are not only poor role models for children, but smoking also dramatically affects children’s health [33,34]. More than 93% knew that smoking causes tooth discoloration and bad breath, and 76.6% knew it causes oral cancer. Similar results were confirmed in a study conducted in Saudi Arabia in 2020 [35].

When asked questions about the health effects of secondhand smoke, the respondents showed below-average knowledge. The most significant proportion of respondents was aware of the harmful effects of secondhand smoke concerning lung cancer (66.2%) and cardiovascular disease (56.6%). The lowest scores were found for questions about the association between smoking and cognitive deficits (51%) and ear infections in children (55.8%). Medical students in Saudi Arabia had a slightly better level of knowledge than the students in this study. Additionally, in that study, ear infections in children (28.1%) and cognitive deficits (47.8%) were the least-known health risks of secondhand smoke [15]. Inadequate knowledge about the harmfulness of passive smoking compared to active smoking was also confirmed among students in Poland [36].

In this survey, the gender of the respondents did not affect knowledge, and the number of active tobacco product users was the same in both genders. The opposite was shown in a Nigerian study, which found that there were more male smokers and that males had better knowledge levels. The higher percentage of males could be due to the social acceptance of smoking among males, a sense of maturity (a symbol of masculinity), and peer influence. The lower prevalence of female smokers could be due to family values, cultural norms, or religion [37]. In contrast to gender, respondents’ age and the time they started using tobacco products influenced their knowledge. Older respondents had better knowledge, as did those who started smoking later, probably because they had more time to become informed and were aware of more advertisements about the harmful effects of smoking. Our study found that most respondents started smoking in high school (70.9%) and college (17.9%). Similar results were obtained in a study in Portugal, where most current smokers started smoking before 17 years (61.4%) [9].

Considering the association between knowledge and the desire to quit smoking, this study shows that respondents who intend to quit using tobacco products know more than those who do not. The positive association between the intention to quit smoking and knowledge of health risks has been confirmed in studies from Iraq, China, and India [21,22,38]. There is ample evidence that well-designed health warnings and messages can be effective interventions to communicate health risks and increase tobacco users’ motivation to quit smoking [39]. In the present survey, most tobacco users continued to use them despite being aware of the health warnings on their packs (81.7%) and being educated about the harmful effects of tobacco use (91.7%). Additionally, they had inadequate knowledge about the harmful effects of smoking. Most respondents (40.4%) believe that there would be fewer smokers if the price of products were higher, and 63.8% believe that smoking should be banned in public places. A high proportion of the Croatian population facing health warnings has been previously confirmed, but without demonstrating a significant impact on smokers’ attitudes and habits [40].

In contrast to health warnings on cigarette packaging, there are also marketing strategies that portray the tobacco industry in a positive light. Restricting tobacco company marketing and sponsorship is a practical component of tobacco control worldwide [41]. Most respondents in our survey (48.1%) had seen tobacco product advertisements or promotions in stores in the past 30 days. Tobacco heater advertisements are primarily targeted at young people. Advertising is widespread, especially online and on social media platforms like Instagram. Social media user behavior analysis has shown that content related to tobacco heating products (e.g., photos showing how to use the device) is frequently shared. All these activities may increase the number of users and the social acceptance of tobacco [21].

Launching more educational campaigns and creating a curriculum in all educational institutions that includes information about tobacco and its harmful effects on health is also recommended. In addition, banning the sale of tobacco and tobacco products on and off campus could be beneficial, as it would help reduce the number of deaths and illnesses caused by smoking [42]. It is important to emphasize the significance of right information and adequate knowledge in changing behavior. In fact, many studies hold that information is important but does not change behavior. In other words, having knowledge is good, but behavior change involves other cognitive and social factors. The data could be helpful for educational institutions to better understand students’ knowledge and promote better education of students through various lectures or workshops [43,44].

## 5. Limitations 

This survey has several limitations. Firstly, it is a cross-sectional study from which causal inferences cannot be drawn. Secondly, the study was conducted with students from the one university in southern Croatia (only one geographical area of Croatia); including other cities would likely yield different results. Therefore, it is recommended that a study be conducted on the student population throughout Croatia and that students be educated about the dangers of tobacco products. Thirdly, university students are not representative of young people in general, and sociodemographic variables and health risk behaviors may differ in other populations. Fourthly, this survey includes only voluntary responses (online survey) and convenience sampling, which could affect sampling bias and generality. Another limiting factor of this study was that a higher percentage of women than men participated in the survey. This could be due to the fact that women are more involved in social activities and are more willing to answer a research survey, since study was restricted to Internet users [45]. It should also be noted that a specially designed questionnaire was used in this survey. In contrast, in the future, validated questionnaires, such as the one from the Institute for Global Tobacco Control, should be used to allow easy comparison of the data of other studies [46].

## 6. Conclusions

The results of this study provide information about the current insufficient knowledge of southern Croatia university students about tobacco products and their harmful effects on general and oral health. Respondents are well aware of the harmful effects of active smoking on health. However, they are not aware of the harmful effects of passive smoking and the differences between different tobacco products. Understanding how young people perceive the harmfulness of tobacco use can contribute to the understanding of the prevalence of tobacco consumption and the development of preventive educational interventions during schooling.

## Figures and Tables

**Table 1:** Demographic characteristics of respondents according to the average score of knowledge about tobacco products and their adverse effects (*n* = 1184).

Characteristics	Categories	Frequency (*n*)	Percentage (%)
Gender	Female	857	72.3
	Male	327	26.7
Age	18–22	723	61.0
	23–25	409	34.5
	≥25	52	4.5
Study field	Biomedical science	230	19.4
	Social sciences	411	34.7
	Technical sciences	162	13.7
	Humanities sciences	154	13.0
	Natural sciences	105	8.9
	Biotechnical science	122	10.3
Year of study	1st year	287	4.2
	2nd year	190	16.0
	3rd year	225	19.0
	4th year	215	18.2
	5th year	181	15.3
	6th year	86	7.3
Family members-healthcare employees	Yes	272	23.0
Family’s monthly budget	Below average	83	7.0
	Average	653	55.2
	Above average	448	37.8

Data are presented as numbers and percentage.

**Table 2:** Characteristics of respondents related to tobacco use behavior based on mean score for knowledge of tobacco products and their harmful effects (*n* = 1184).

Characteristics	Categories	Frequency (*n*)	Percentage (%)
Tobacco product use	Former user (not smoked in the last 28 days)	66	5.6
	Current	357	30.1
Type of tobacco product use among current users of tobacco products	Cigarettes	266	74.5
	E-cigarettes	28	7.9
	Heated tobacco products	63	17.6
Started using tobacco products among current users of tobacco products	Elementary school	40	11.2
	High school	253	70.9
	University	64	17.9
Duration of tobacco product usage among current users of tobacco products	≤1/year	20	5.6
	1–2/years	47	13.2
	3–5/years	169	47.3
	≥5/years	121	33.9
Who knows that you use tobacco products among current users of tobacco products	Family	4	1.1
	Friends	110	30.8
	Family/friends	242	67.8
	No one	1	0.3
Want to quit use of tobacco products among current users of tobacco products	Yes	204	57.1
Family members use tobacco products	Both parents	203	17.1
	Father	196	16.5
	Mother	175	14.8
	Grandparents	21	1.8
	Sibling(s)	142	12.0
	Nobody	447	37.8
Familiar with smoke-free devices	Yes	978	82.6
Smoke-free devices are a better alternative to traditional smoking	Yes	555	46.9
Familiar with the difference between cigarettes, e-cigarettes, and tobacco heating devices	Yes	590	49.8

Data are presented as numbers and percentage.

**Table 3:** Respondents’ opinion on exposure to health warnings and tobacco product advertising messages in relation to the average level of knowledge about tobacco products and their harmful effects (*n* = 1184).

Question	Categories	Frequency (*n*)	Percentage (%)
Did you notice messages in the media against smoking during the last 30 days?	Yes	434	36.7
Did you notice tobacco advertisements or its promotion in shops during the last 30 days?	Yes	570	48.1
Did you notice someone using tobacco on TV, videos or films during the last 30 days?	Yes	859	72.6
Do you notice health warnings on cigarette packaging?	Yes	967	81.7
Did you learn about the dangers of using tobacco products during schooling?	Yes	1086	91.7
There would be less smokers if?	The price increases	478	40.4
	Young people were educated	311	26.3
	Health workers informed the public about the adverse effects of smoking more frequently	250	21.1
	Marketing and advertising were limited	145	12.2
Smoking should be banned in closed public spaces?	Yes	755	63.8

Data are presented as numbers and percentage.

**Table 4:** Unadjusted and adjusted odds ratios for the associations of participants’ characteristics with knowledge of tobacco products and their harmful effects and knowledge of adverse health effects of tobacco.

Independent Variable	Categories	Knowledge of Tobacco Products and Their Harmful Effects		Knowledge of Adverse Health Effects of Tobacco	
		Unadjusted OR (95% CI)	Adjusted OR (95% CI)	Unadjusted OR (95% CI)	Adjusted OR (95% CI)
Gender	Male	1		1	
	Female	1.10 (0.85–1.43)	1.11 (0.83–1.47)	1.28 (0.99–1.66)	1.25 (0.94–1.67)
Age (years)	18–22	1		1	
	23–25	1.58 (1.24–2.03) ^c^	1.27 (0.97–1.65)	1.60 (1.24–2.06) ^c^	1.23 (0.92–1.61)
	≥25	2.00 (1.12–3.59) ^a^	1.81 (1.01–3.41) ^a^	1.67 (0.92–3.045)	1.69 (0.90–3.17)
Study field	Biomedical science	1		1	
	Technical sciences	0.26 (0.17–0.40) ^c^	0.28 (0.18–0.45) ^c^	0.12 (0.07–0.21) ^c^	0.15 (0.09–0.25) ^c^
	Social sciences	0.33 (0.23–0.47) ^c^	0.33 (0.23–0.48) ^c^	0.14 (0.09–0.22) ^c^	0.15 (0.09–0.24) ^c^
	Humanities sciences	0.21 (0.13–0.34) ^c^	0.23 (0.14–0.37) ^c^	0.13 (0.07–0.22) ^c^	0.14 (0.08–0.25) ^c^
	Natural sciences	0.32 (0.20–0.53) ^c^	0.37 (0.23–0.61) ^c^	0.16 (0.09–0.28) ^c^	0.18 (0.10–0.33) ^c^
	Biotechnical science	0.26 (0.16–0.42) ^c^	0.27 (0.17–0.44) ^c^	0.16 (0.09–0.27) ^c^	0.16 (0.09–0.29) ^c^
Family members/healthcare employees	Yes	1		1	
	No	1.04 (0.93–1.17)	1.01 (0.76–1.36)	0.69 (0.52–0.92) ^a^	0.86 (0.64–1.18)
Family’s monthly budget	Below average	1		1	
	Average	0.77 (0.48–1.22)	0.79 (0.49–1.28)	0.80 (0.50–1.28)	0.81 (0.49–1.33)
	Above average	1.19 (0.74–1.19)	1.11 (0.67–1.84)	1.28 (0.79–2.08)	1.20 (0.72–2.01)
Tobacco product use	Never	1		1	
	Former user	1.96 (1.16–3.33) ^a^	1.90 (1.09–3.31) ^a^	2.11 (2.18–3.79) ^a^	2.02 (1.09–3.73) ^a^
	Current	1.30 (1.01–1.68) ^a^	1.41 (1.08–1.84) ^a^	0.93 (0.72–1.20)	1.02 (0.77–1.34)

Note: ^a^ *p* < 0.05.; ^c^ *p* < 0.001. Reference knowledge level category is “poor”. OR: odds ratio; 95% CI: 95% confidence interval.

**Table 5:** Unadjusted and adjusted odds ratios for the associations of participants’ characteristics with and health risks of secondhand smoke and the knowledge of tobacco products.

Independent Variable	Categories	Knowledge of Health Risks of Secondhand Smoke		Knowledge of Tobacco Products	
		Unadjusted OR (95% CI)	Adjusted OR (95% CI)	Unadjusted OR (95% CI)	Adjusted OR (95% CI)
Gender	Male	1		1	
	Female	1.06 (0.82–1.37)	1.01 (0.83–1.46)	1.04 (0.79–1.27)	0.92 (0.68–1.26)
Age (years)	18–22	1		1	
	23–25	1.87 (1.46–2.40) ^c^	1.60 (1.23–2.08) ^c^	1.03 (0.79–1.34)	0.99 (0.47–1.32)
	≥25	0.91 (0.52–1.61)	0.85 (0.47–1.53)	1.70 (0.86–3.37)	1.22 (0.60–2.54)
Study field	Biomedical science	1		1	
	Technical sciences	0.38 (0.25–059) ^c^	0.44 (0.28–0.68) ^c^	0.57 (0.37–0.87) ^b^	0.52 (0.33–0.83) ^b^
	Social sciences	0.39 (0.27–0.55) ^c^	0.45 (0.31–0.65) ^c^	1.26 (0.88–1.80)	1.17 (0.79–1.70)
	Humanities sciences	0.31 (0.19–0.49) ^c^	0.38 (0.23–0.61) ^c^	0.85 (0.53–1.35)	0.75 (0.45–1.23)
	Natural sciences	0.37 (0.23–0.60) ^c^	0.40 (0.24–0.66) ^c^	1.19 (0.71–2.01)	1.22 (0.71–2.10)
	Biotechnical science	0.23 (0.14–0.37) ^c^	0.25 (0.15–0.40) ^c^	0.89 (0.55–1.43)	0.86 (0.52–1.41)
Family members/healthcare employees	Yes	1		1	
	No	0.76 (0.58–1.00)	0.86 (0.64–1.15)	0.70 (0.58–1.08)	0.81 (0.58–1.11)
Family’s monthly budget	Below average	1		1	
	Average	0.94 (0.59–1.49)	0.88 (0.54–1.53)	0.30 (016–0.57) ^c^	0.30 (0.16–0.58) ^c^
	Above average	1.06 (0.66–1.69)	0.92 (0.56–1.51)	0.47 (0.24–0.91) ^a^	0.44 (0.22–0.87) ^a^
Tobacco product use	Never	1		1	
	Former user	1.40 (0.84–2.35)	1.32 (0.77–2.27)	1.08 (1.64–1.84)	1.05 (0.66–1.82)
	Current	0.93 (0.72–1.20)	0.99 (0.76–1.29)	3.21 (2.23–4.43) ^c^	3.17 (2.17–4.24) ^c^

Note: ^a^ *p* < 0.05.; ^b^ *p* < 0.01.; ^c^ *p* < 0.001. Reference knowledge level category is “poor”. OR: odds ratio; 95% CI: 95% confidence interval.

**Table 6:** Gender-specific generalized linear model regression for estimating the odds ratio and 95% confidence interval for knowledge about tobacco products and their harmful effects.

Independent Variable	Categories	Male		Female	
		OR (95% CI)	*p*-Value	OR (95% CI)	*p*-Value
Age (years)	18–22	1		1	
	23–25	1.12 (0.67–1.87)	0.147	1.60 (0.77–3.34)	0.114
	≥25	2.28 (0.74–6.94)	0.646	1.29 (0.93–1.79)	0.206
Study field	Biomedical science	1		1	
	Social sciences	0.28 (0.12–0.61)	≤0.001	0.34 (0.22–0.52)	≤0.001
	Technical sciences	0.27 (0.12–0.60)	≤0.001	0.24 (0.13–0.44)	≤0.001
	Humanities sciences	0.26 (0.06–1.07)	0.061	0.22 (0.13–0.37)	≤0.001
	Natural sciences	0.15 (0.05–0.48)	≤0.001	0.47 (0.26–0.83)	0.009
	Biotechnical science	0.08 (0.03–0.31)	≤0.001	0.32 (0.19–0.55)	≤0.001
Family members/healthcare employees	Yes	1		1	
	No	0.51 (0.28–0.94)	0.030	1.23 (0.90–1.79)	0.175
Family’s monthly budget	Below average	1		1	
	Average	1.55 (0.41–5.85)	0.512	0.75 (0.44–1.27)	0.282
	Above average	1.81 (0.47–7.01)	0.385	1.16 (0.66–2.03)	0.608
Tobacco product use	Never	1		1	
	Former user	1.38 (0.44–4.27)	0.571	2.11 (1.10–4.04)	0.023
	Current	1.32 (0.77–2.24)	0.300	1.41 (1.05–1.99)	0.021

Reference knowledge level category is “poor”. OR: odds ratio; 95% CI: 95% confidence interval.

## Data Availability

The data that support the findings of this study are available upon request from the authors.

## Supplementary Materials

The following supporting information can be downloaded at: https://www.mdpi.com/article/10.3390/healthcare11050771/s1, Table S1: The frequency distribution (%) of respondents’ answers about tobacco products and their adverse effects on human health.
