# Supplementary material for: Prevalence of Use and Knowledge about Tobacco Products and Their Harmful Effects among University Students in Southern Croatia
Source: Healthcare (Basel). 2023 Mar 6;11(5):771. doi: 10.3390/healthcare11050771 (PMC10001000; doi:10.3390/healthcare11050771)
Supplement: Supplementary file 1 [file healthcare-11-00771-s001.zip › healthcare-2074913-supplementary.pdf]

Supplementary file

Table S1. The frequency distribution (%) of respondents' answers about tobacco products and their adverse effects on human health

Question	Answer	
	No	I don't know
Smoking increases the risk of developing cancer (lungs, liver, pancreas, kidney, leukemia, throat…).	0.8	8.6
Smoking causes heart and blood vessel diseases.	1.5	13.0
Smoking causes lung diseases (chronic obstructive lung disease, chronic bronchitis).	1.4	9.1
Smoking is linked to cognitive deficiencies.	8.8	42.1
Smoking is linked to strokes.	4.6	26.5
Smoking causes premature skin aging.	2.4	19.7
Smoking causes eye diseases (increases the risk for age degeneration of the macula and cataract).	5.5	42.9
Smoking decreases bone density which means a higher risk for broken bones, including hip fracture.	9.6	51.5
Smoking increases the risk of rheumatoid arthritis.	7.3	51.2
Smoking increases teeth staining and bad breath.	1.2	5.7
Smoking causes changes in taste and smell.	5.1	22.7
Smoking causes oral carcinoma.	1.8	21.6
Smoking increases the risk of periodontal diseases - periodontitis and tooth loss.	3.0	19.0
Smoking during pregnancy affects the child's health.	1.4	6.9
Exposure to passive smoking causes lung cancer.	5.9	27.9
Exposure to passive smoking causes heart and blood vessels diseases.	7.8	35.6
Exposure to smoking is linked to cognitive deficiencies.	11.7	51.0
Exposure to passive smoking causes ear infections in children.	12.6	55.8
Exposure to passive smoking is linked with children's allergies.	7.0	45.8
Exposure to passive smoking is linked with asthma in children.	4.5	36.9
A cigarette burns at 800 degrees.	6.7	64.3
A complex mixture called smoke occurs during cigarette burning.	1.5	14.7
Tobacco heating devices use a tobacco insert.	2.6	45.4
During the heating of the tobacco insert smoke is not produced but aerosol.	3.5	56.7
Tobacco heating devices use real tobacco.	11.4	57.4
By heating tobacco 50% less of the harmful substances are released.	5.6	60.4
E-cigarettes use fluid in which nicotine was synthetically added.	3.1	40.0
Data are presented as percentage.		
